# Participant perspectives on a person-centred care model for mobile individuals in HIV care from the SEARCH Dynamic Choice Treatment trial in rural Kenya and Uganda

**DOI:** 10.1371/journal.pone.0356605

**Published:** 2026-09-03

**Authors:** Jason Johnson-Peretz, James Ayieko, Lawrence Owino, Cecilia Akatukwasa, Anjeline Onyango, Fred Atwine, Titus O. Arunga, Colette Aoko, Laura B. Balzer, Moses R. Kamya, Edwin Charlesbois, Jane Kabami, Maya L. Petersen, Gabriel Chamie, Diane V. Havlir, Elijah Kakande, Carol S. Camlin

**Affiliations:** 1 Division of Prevention Sciences, University of California, San Francisco (UCSF), San Francisco, California, United States of America; 2 Kenya Medical Research Institute (KEMRI), Kisumu, Kenya; 3 Infectious Diseases Research Collaboration, Kampala, Uganda; 4 University of California, Berkeley, Biostatistics, Epidemiology, and Computational Precision Health, Berkeley, California, United States of America; 5 Department of Medicine, Makerere University College of Health Sciences, Kampala, Uganda; 6 University of California, San Francisco (UCSF), Medicine, San Francisco, California, United States of America; University of Zimbabwe, Faculty of Medicine and Health Sciences, ZIMBABWE

## Abstract

**Introduction:**

Mobile populations experience difficulties maintaining antiretroviral therapy (ART) adherence and HIV care engagement. We sought to explore participant perceptions of a patient-centred model aimed at enhancing care engagement through ‘dynamic choice’ treatment services for mobile individuals.

**Methods:**

We conducted 23 qualitative interviews with participants (N = 18) and healthcare providers (N = 5) from eight communities in southwest Uganda and western Kenya enrolled in a dynamic choice intervention that provided six-month and community ART refills; a “travel pack” with a travel checklist, discreetly packed ART, and an emergency supply of ART; phone access to a mobility coordinator; and facilitated transfers to distal clinics. Interviews explored participant life and mobility contexts, intervention experiences and preferences (as recipient or provider), and patient-provider relations. We employed a framework analysis of selected codes to identify emergent themes around intervention components and recommendations for improvement.

**Results:**

Participants liked all options, the travel pack being most preferred. Longer times between refills allowed participants to concentrate on ART adherence, rather than on managing clinic appointments in the context of uncertain mobility. Viral load counselling was appreciated for explaining the relationship between medication adherence, viral load, and opportunistic infections. Poverty and food insecurity remain obstacles to ideal ART adherence.

**Recommendations:**

Participants suggested scaling up these support services to the whole community, incorporating services to treat additional diseases, and food or employment support to help improve overall health outcomes. Care providers also recommended strengthening supply chains and deploying electronic medical records country-wide to facilitate care for mobile individuals.

## Background

To end the HIV epidemic, addressing the needs of people living with HIV who struggle to engage in HIV care and treatment is crucial. In health systems designed for stable residence, mobile individuals face unique challenges to care engagement and antiretroviral therapy (ART) adherence [1–5]. Studies have shown that travel and lack of contact with providers are common reasons for dropping out of HIV care [5–7]. While some mobile people with HIV (PWHIV) engage in care, most experience challenges to adherence and to remaining virally suppressed [8,9]. In a prior trial, we showed that patient-centred care through choice of treatment support options tailored for mobile populations improved retention in care and ART possession over 48 weeks [8]. Since the needs of mobile PWHIV can change over time, we described this as a dynamic choice treatment model of delivery.

In low- and middle-income countries (LMICs), the term ‘mobile individuals’ encompasses people who migrate to change their residence within or across national boundaries, either temporarily or permanently, for economic opportunity or as a result of economic shocks or other drivers (such as civil unrest). The category also includes those who do not change residences but spend periods of time away from home to travel for work or to seek employment, or for reasons such as caregiving, care-seeking, or education [10–12]. This mobility can involve both predictable travel and unplanned trips over which individuals have little control. Men’s and women’s differing labour market opportunities and social role expectations drive differences in their patterns of movement, types of destinations, and consequences of their mobility, which in turn influence HIV risks and outcomes [7,13–16]. For example, while both men and women often travel to look for work, men travel for longer distances and time periods, whereas women return more frequently to their primary residences [12,17,18]. Women’s more limited formal sector labour opportunities mean that many supplement their earnings with commercial and transactional sex work [19,20].

Patient-centred care is one way to follow patient preferences and improve patient-provider relationships to address the unmet needs of populations such as those who are highly mobile. While differentiated service delivery (DSD) models focus on addressing structural barriers to care engagement, patient-centred care can reach all populations because it is concerned with addressing patients’ needs for care that affirms their agency and dignity and is responsive to their cultural values, understanding the whole person. Patient-centred care recognises the heterogeneity of patients in order to apprise individuals accurately about their levels of risk, while working with them to find the most appropriate means to address their current circumstances [21]. Additional components of patient-centred care include friendly providers who cultivate positive patient-provider and patient-clinic relationships and reduce stigma, distinguish between those who need intensive counselling and those who can engage with streamlined care, and enhance knowledge of HIV and ART as necessary [22]. Such relationships are fostered through empathy and communication skills sensitive to the life stage or circumstances of the patient [23–25]. These approaches that establish rapport, broach sensitive topics, or engage in motivational interviewing can help promote greater engagement in patients’ own healthcare, while potentially decreasing physician burnout [24]. Patient-centred care also aligns with the values espoused by ethical personalism as articulated by several African [26–29] and European [30–34] philosophers. (Personalism is a philosophical ethic opposing the commodification of persons and their alienation through both consumerism and an individualism that leaves people to their own devices [34].)

The SEARCH-SAPPHIRE Dynamic Choice Treatment (DCT) trial sought to assess a patient-centred model serving mobile PWHIV [8]. The DCT trial placed the person in care and their needs or preferences at the centre of the intervention. Results from the main trial indicated that receiving the intervention led to significantly higher patient retention in HIV care and ART possession, as compared to the standard-of-care but there was no significant difference in viral suppression [8]. This paper therefore seeks to describe participant and provider perceptions of the intervention in order to identify what may have led to higher patient engagement in care while also presenting participant recommendations for improvement of individual components of that model. Through these approaches, we sought to uncover how much of the intervention is potentially scalable or replicable for health systems in other rural areas where mobility forms a core part of the economic life of the community.

## Methods

### Study context and participating sites

To reduce HIV incidence to below 0.1% through innovative and scalable HIV treatment and prevention strategies and patient-centred care, the SEARCH-SAPPHIRE Study (NCT04810650) deploys a multisectoral strategy drawn from implementation science, precision medicine, and population health to reach “persistent driver” populations. Conducted in 5 rural clinics in western Kenya and southwestern Uganda with high HIV prevalence according to Ministry of Health (MoH) data, the Dynamic Choice Treatment study was one of several trials in the first phase of the study. Following results gathered from ranked choice surveys among a subset of our population in rural western Kenya, the SEARCH Collaboration designed a differentiated, patient-centred clinical care model intervention to address the needs of mobile participants with HIV. We defined mobility as 2 or more weeks with nights spent away from home and outside the community during the 12 months before enrollment.

Intervention components in this two-arm study have been described in more detail in the primary paper [8]. Briefly, its seven components included provision of a “travel pack” containing a travel checklist (with mobility coordinator phone number) to remind participants of what to pack when travelling, discreet ART packing options, and an emergency supply of 14 extra ART pills; a mobility coordinator who would ask participants about their travel plans at each visit and coordinate care with them accordingly; “hotline” phone and SMS access to reach the mobility coordinator as needed; extended refills up to 6 months; community-based delivery of medication for persons unable to return to clinic; assistance in navigating out-of-community ART refills; and transfers of care to out-of-community HIV clinics (when out-migrating without a plan to return) [8]. Viral load counselling for participants was also a core component of the SEARCH-SAPPHIRE patient-centred model [22,35]. Control arm participants received usual care and follow-up delivered by the Ministries of Health in the respective countries.

### Data collection

A gender-balanced team (3 men, 2 women) of trained qualitative researchers who were native speakers of the local languages DhoLuo, English, Runyankole, and Swahili administered audio-recorded, in-depth semi-structured interviews to N = 18 purposively selected participants who received the Dynamic Choice Treatment intervention. The selection was balanced by sex, livelihood or profession, and community; recruitment began 13 October 2021 and interviews occurred between 24 January and 05 April 2022. We also interviewed a selection of providers of HIV treatment care (N = 26; 5 were DCT providers, including 3 mobility coordinators specifically; all male) during the same period. After consenting the participants, we ensured confidentiality by conducting interviews in private and comfortable locations convenient for the interviewees. Interviews with both groups explored patient-participant life and mobility contexts, experiences with the intervention (either as a recipient or provider), preferred services in the intervention, patient-provider interaction, and discussions with peers about HIV, HIV prevention, and HIV stigma. Table 1 presents the demographic information of our qualitative cohort.

**Table 1:** Participant Demographics in N(%)*.

Clients	N = 18
**Country**	18 (100%)
Kenya	8 (44%)
Uganda	10 (56%)
**Sex**	
Female	10 (56%)
Male	8 (44%)
**Age**	
Range	22 - 49 yrs
Mean (Median, Mode)	35.5 (35, 35)
**Arm**	
Control	4 (22%)
Intervention	14 (78%)
**Providers**	N = 5
Mobility Coordinators	3 (60%)
Study Nurse	1 (20%)
Research Assistant	1 (20%)
**Country**	5 (100%)
Kenya	2 (40%)
Uganda	3 (60%)
**Sex**	
Female	0 (0%)
Male	5 (100%)
**Age**	
Range	34 - 47 yrs
Mean (Median)	39.2 (36)

*The top set of demographics pertains to patient-clients, the bottom section to the providers interviewed for this study.

### Data analysis

The interviews were transcribed and translated into English, then uploaded into Dedoose software [36]. Using the R-EIGHT method for globally dispersed qualitative research teams, the team generated a set of inductive codes that were then compared and combined with an *a priori* codebook derived from the theory-informed IDI topic guides [37]. This gave us a final codebook reflecting both detailed emergent codes rooted in observations derived from culturally informed, inductive analysis of our data and broad concepts derived from theory and prior research questions. An eight-person team (LO, AO, CA, FA, IM, TA, JL, JJP) then coded the transcripts with this codebook. Following coding, we deployed a framework analysis of selected codes to identify emergent themes that answered our primary research questions [38–41]. We exported coded excerpts from the following broad codes: mobility, ART and adherence, viral load literacy, service perceptions, and recommendations. (The specific codes are in Table S1). These excerpts included data from both patient-client and provider participants.

### Ethical approval and informed consent

The University of California San Francisco Committee on Human Research, the Ethical Review Committee of the Kenya Medical Research Institute, the Makerere University School of Medicine Research and Ethics Committee and the Uganda National Council for Science and Technology all approved this study (UCSF 20−32144; KEMRI No. 4173; UNCST No. HS1239ES; SOMREC No. 2020−29). All study participants provided written and informed consent to participate in interviews.

## Results

Below, we examine participant contexts and experiences with the intervention before presenting participant suggestions for improvement or expansion of services.

### Perception of intervention components

#### Travel pack.

The travel pack option – provided in an unmarked wallet for men and a handbag for women – was incredibly popular among participants. Its popularity was partly a result of the pack’s versatility. Several participants used it for ID cards, money, and medication, as well as study-provided discreet packaging for medication. It was also a way to keep everything necessary for care engagement and ART adherence in one place in case of abrupt travel: “*They put the forms, a phone number and the extra fourteen pills and 20,000 shillings for transport for that day when I received the pack. I was also given a box-like object with compartments for putting in my tablet*.” (49 y.o. female, Uganda) The pack also eased stigma associated with taking medication when travelling:

“Well, this wallet is very important for me. In case I want to travel, I will make some kind of preparations. First, I would take my medication and, after placing those medications in a drug envelope, then I put it in my wallet, depending on the number of days I am going to stay away. During that period when I am away, I would just remove my pill as if I am getting some money from the wallet and take it. This has eased my work because if you carry those medications in something else apart from the wallet – let us say the bottle – then people around you become suspicious of you. But with the wallet, they would think I want to count my money, yet I am taking my pills (laughter).” (49 y.o. male, Kenya)

The benefit of keeping everything together was useful even for less mobile participants, who mainly used the travel pack when coming to the health facility for medication refills or for last-minute or far-away travel. A provider summed up the popularity of the travel packs and wallets:

“Actually, all men have opted to have their packs. Some women have preferred men’s packs <i.e., wallets> and not women’s because men’s packs are smaller… The men’s travel pack is something that can go into your pocket and some women have opted to go for that than to go for their packs because of the size. …Even when you find that someone has not travelled, they use the travel packs frequently. Someone would prefer to first open the travel pack and use the drugs and later replace them instead of opening the usual drug tins. So, I have not heard of the challenges or people complaining about them <but rather> people embracing them” (34 y.o. Study Nurse, Uganda)

In fact, multiple participants appreciated the extra twelve to fourteen pills in their bottles to serve a stop-gap measure in case of unexpected travel or delay in returning to the clinic and said it relieved them of stress in case of unexpected circumstances. The only concern a few participants expressed is that if the packs become widespread (i.e., given to all people in the area diagnosed with HIV), people would understand that they were given only to people receiving ART.

#### Mobility coordinators and mobility conversations.

A cornerstone of the intervention was asking participants about their travel plans; several other components depended on the response to this question. Participant experiences were tied to different types of mobility, as “*some are mobile on economic bases while some on social grounds*.” (35 y.o. Mobility Coordinator, Kenya)

Economic reasons for mobility included occupations such as fishing, trading, or work in the transport and construction sectors, as well as searching for employment. Social reasons included marriage, bringing family members to hospital, visiting friends, and funerals. Time away from home ranged from a day or two when travelling nearby, to between three and five months for construction jobs or bar work, to longer journeys in both time (more than five months) and distance (crossing international borders) for traders. Typically, shorter-term mobility was more abrupt, while longer-term travel was pre-planned. A few participants did not consider themselves mobile, or had their previous mobility curtailed due to pregnancy, illness, or injury (and potentially age).

Overall, patient-clients liked being asked about their mobility plans because it gave them a chance to work with the provider and strategise support for ART adherence as expressed by participants from two different sites:

“<Being identified as mobile> helped me a lot because at times I would want to travel yet couldn’t come and pick up medication before the next appointment.” (35 y.o. female, Uganda)

“I loved the idea since in the past, we would not be able to get a chance to talk with the provider and for the provider to understand our challenges. At the moment, I can come and talk to him, and he would ask, ‘*How many days are you going to take outside this community? Take this; I am sure you will be back by this date*.’” (31 y.o. female, Kenya)

One provider emphasized the importance of being specific when making this inquiry:

“It is also upon the provider to make it clear that a ‘travel’ can even take a day. Sometimes, when you ask about the travels, they think that they need to be carrying bags and travel to Nairobi or Kisumu. Yet they might travel to the mainland <from Mfangano Island> or Rusinga Island and spend a day or a night. You have to remind them about this all the time for them to inform you so that you can support them during their travels.” (36 y.o. Mobility Coordinator, Kenya)

Participants remarked that such openness with their providers around travelling was new to them, and some quickly adapted to the change, incorporating planning medication and informing the providers into their trip plans.

#### Viral Load Notification and counselling.

Although providers and staff are trained on viral load counselling, the mobility coordinators also had the opportunity to fill gaps and reinforce participant knowledge around viral load. Confusion sometimes persisted about the difference between CD4 levels and viral load; as one participant admitted, “*I didn’t understand whether all worked the same way that is the CD4 being low and also viral load low or otherwise*” (49 y.o. female, Uganda), and some participants even thought it was good to have both a high CD4 count and a high viral load. Nonetheless, a mobility coordinator explained that,

“Initially, the importance of viral load was not emphasised; the service provider would take the blood sample and inform the patient that the results were not looking good. The patients did not understand what was being tested. Today, they are more informed about the viral load results; they know how to interpret the results and what it means and know what to do to improve.” (36 y.o. Mobility Coordinator, Kenya)

Reviewing viral load results with patients and counselling them on what it meant helped providers create strategies with their patients to support ART adherence: “*When they give me my result – let us say <hypothetically> 1000 or 2000 copies – they will have to explain to me why it is at that level. They will offer me counselling about the reasons why it is high and what I should do in order to reduce my viral load level.*” (49 y.o. male, Kenya) Meanwhile, providers felt viral load counselling fostered greater openness with patients. “*Viral load and adherence counselling has been so important to most patients’ lives, in addition to the strong rapport between the patients and us; this is so because, despite all odds, they feel cared for*” (35 y.o. Mobility Coordinator, Kenya). Once participants understood the relationship between adherence and viral load, they reported being motivated to adhere to achieve viral suppression, and the only drawback mentioned was anxiety if it was high.

#### Phone contact and streamlined care*.*

Participants appreciated the quicker service at clinic and pharmacy, as well as the friendly providers who would conduct follow-up calls, both of which were new to many participants.

“This provider is good because he calls me two days before my appointment. If he does not see you in the clinic on that day and you tell him that you are at home or in the market, he will carry the medication and give it to you privately. This helps because those of us who sell *omena* <a small Lake Victoria fish> might be very busy until after dark, and you might only remember your appointment at your pill time. It has helped us.” (35 y.o. female, Kenya)

Phone contact with providers was new to some participants, who felt reassured that they would still receive HIV care if they woke up sick. Several participants used the provider contacts for assistance. In the context of financial constraints, while phone contact was appreciated, some observed that phone contact with airtime would be better, and a provider noted the challenge of patients sharing phones and giving someone else’s phone number as the contact.

#### Longer refills*.*

The local MoH clinics had begun extending refills from between one and three-month long refills per visit to six-month refills during the COVID pandemic to reduce congestion at the facilities. Yet longer refills also helped save on cost of transport, eased work schedule conflicts and the stigma of frequent clinic visits, and permitted participants to focus on adherence rather than on clinic appointments.

“Previously, I used to be given one month, but now I am given three months, and this helps me in saving on transport to come to the hospital. I was later given five months. …One is not under pressure to come every other time for their clinic appointment so one concentrates on taking their medication till the next appointment.” (35 y.o. female, Uganda)

Longer refills meant fewer clinic appointments and less community speculation about why some participants were ‘always’ at the facility, allowing participants to avoid the stigma associated with recurrent clinic visits. *“(chuckles)…you know that when we walk around the community, people say, ‘*So and so is always going to the hospital.’ *The people within the facility love that, but within the community, people say, ‘*She keeps going to the hospital, what is she going to get*?’ It has helped me with that*.” (31 y.o. female, Kenya) In this way, the longer refills helped some participants avoid the (self-)stigma that resulted from monthly visits to the clinic, potentially facilitating greater adherence.

#### Synergy between mobility conversations, phone contact, and multi-month refills.

The longer refill option plus sharing mobility plans meant that if a participant had a journey, they felt able to call the providers, state how much ART remained in their current prescription, and the providers would top up the remainder. Longer refills depended in part on mobility, but also on demonstrated adherence patterns.

“When I started care, I was coming to the hospital every month. I think they also assess your commitment and persistence to care; if you are coming for your appointment without missing, then they adjust your schedule. This made them give me a refill every two months as they continued to assess whether I could obey the two-month appointment schedule. Then currently, they are giving me after three months because I usually keep my scheduled clinic appointment date. They are happy with me.” (49 y.o. male, Kenya)

The usual length of refill was three months, but some more mobile persons had longer refills. Some participants felt those less engaged in care might forget to return for their refills. Yet longer refills also came with encouragement to contact providers if any challenge should arise, as one participant noted: “*I was told to be reporting to them any challenge that I may be experiencing when I have a long-term stock of the drugs, either through a phone call or I may physically come to the health facility.*” (29 y.o female, Kenya) A more important drawback, however, was mentioned by one participant who related a time when the clinic was short on stocks of ART, compromising their usual ability to give out longer refills:

“I remember there was a time I was given a refill lasting for a month, and I asked, ‘Why are we given such short refills?’ The then healthcare providers said, ‘*There are few doses of the drugs in our stock and thus, we are giving such so that all of you may benefit*.’ At times, we could be given a refill lasting for only three weeks, and it may be this time when you have no money for fare and the children are also out of school due to lack of school fees. … This was so devastating.” (38 y.o. female, Kenya, beginning virally unsuppressed)

Study providers were careful to indicate the tailored nature of longer refills:

“For those who requested for offsite delivery, now, it will depend on the time we gave. Some we give 6 months or 4 months depending on the condition, or like I told you, for 2 months to those with issues <with adherence>. But someone can’t go beyond 6 months without coming to the clinic, even if they opted for a long refill. So we make sure that at 6 months he comes back within the clinic because we want to fully assess the condition of the patient by the clinician.” (47 y.o. Mobility Coordinator, Uganda)

#### Community ART delivery and other services.

Participants also found off-site delivery of medication to be new and well-liked; in some cases, community delivery of medication also meant blood draws were done at home for viral load testing. Participants liked offsite delivery because it assured them of receiving on-time refills in case they couldn’t make a clinic appointment due to illness: “*The only thing that runs in my mind was that someone can fall sick at any time, and if this happens, whom can I send? Therefore, when the provider told me he could bring me medication at home, I was impressed*.” (49 y.o. male, Kenya)

The few participants who did not use community delivery tended to be in town when their medication refill time approached; for those participants, the longer refills were more beneficial than home delivery. Those who didn’t use this option noted that they had the provider's phone number and could call the clinic in case they needed to make use of the service. The drawback participants mentioned when it came to home delivery was that the providers came only with ART, not medication for other health issues.

With respect to the final two components of the intervention, although the intervention attempted to streamline transfer letter requests and broker care at other clinics, other clinics might have different operating hours or procedures, as one participant discovered when she attempted to access medication at a clinic where she took her mother for care:

“I had some pills to last me for four days… <My provider> suggested going to the ART clinic at the facility where my mother was admitted. She said I should tell her how it goes at that clinic. When I got there, they told me, ‘*We do not work on this day, we work on such and such a day*.’” (22 y.o. female, Uganda)

Facilitating transfers, however, was comparatively easier, and several mobility coordinators described this process:

“What we do is we tell them to find a nearby health facility, then after identifying it, we track and get contacts from the facility and tell them we have our participants who are part of the study… After this, we tell our participant to go on that very day or a specific day and look for X to help them. It has been working, and we haven't failed to link any of our participants who wanted to be transferred out.” (47 y.o. Mobility Coordinator, Uganda)

Despite transferring to a new clinic, some participants still considered the study clinic to be their ‘home’ clinic. Although we did not interview participants who transferred out of the community, mobility coordinators maintained contact with the ‘distal’ clinic to facilitate the participant’s return home:

“I have another patient who receives his refill in <a different> sub-county hospital. I instructed him on how to go about it, and he received his drugs; these people still consider this as their home, and they say that they will come back to the community once in a while. When they come back, we encourage them to let us know so that we can do a review to know how they are doing and what needs to be done.” (36 y.o. Mobility Coordinator, Kenya)

### Participant and provider recommendations

Intervention and control arm participants offered suggestions and recommendations about which services could be improved, which should be continued, and what new services they would like to see added.

#### Continued services.

Participants recommended continuing longer refills and the flexibility of community delivery. Participants advocated continuing viral load testing “*because it helps us a lot”* (36 y.o. female, control arm, Uganda). They appreciated adherence encouragement calls from providers (including the mobility coordinators who monitor participant progress), and wanted their availability for phone consults to continue*.* Providers concurred:

“…You know when you give patients like a six-month refill, we normally give them calls every three months. I didn’t know that even that one would be there and it has been very good because whenever you call a patient and you say that ‘*I am X your health worker trying to find out how you feel*,’ they are like, ‘*I feel very happy, I feel so good to talk to you’*.” (47 y.o. Mobility Coordinator, Uganda)

Participants also wanted mobility conversations to continue:

“Actually, when the provider asked such questions, he just reminds me to be active in care, and he triggers my mind to go to the hospital for my medication. … I always take it positively because he is trying to help me. … I would add that let them continue asking me such questions to keep me awake… (laughter).” (49 y.o. male, Kenya)

As mentioned, the travel packs were very popular, and aside from the men’s packs also being popular with women, the only caution participants expressed was that if the packs are given only to PLHIV then the community will come to know the packs signify someone with HIV. Therefore, a derived suggestion is to make travel packs available to anyone with medication-treated chronic diseases (e.g., diabetes, hypertension).

#### Service improvements.

Most improvement suggestions came from people who were well pleased with the viral load component and wanted more frequent viral load testing, quicker turnaround time, and consistent sharing of results to strategise with the providers around adherence if necessary. “*I would like to know the specific amounts of the viral load so that I can be able to tell how much the viral load has reduced. … I wish they test our viral load every two months such that these two months I have spent while taking ARVs, I should be able to tell the levels*.” (22 y.o. female, Uganda) Participants also suggested even longer refills or a yearly injection, though some paired this with three-month check-in calls to ensure the patient isn’t facing setbacks. One person suggested that the mobility coordinator follow up in person if the participant is unreachable by phone.

Participants also expressed concerns about potential interactions between pharmaceutical medication (e.g., diabetes pills) and ART. Similar concerns related to how diet and ART interact, or how diet can support ART. Participants tended to think about food in relation to how ART works partly because of the context of food insecurity, and this concern often, but not always, came from participants who were food insecure or who also recommended the clinic supply some food provisions. In this context, some participants incorporated such social determinants of health into what would otherwise be a lay explanatory model for how ART works on the body, suggesting that participants form their own ‘extended’ or ‘holistic’ explanations about HIV treatment efficacy beyond ART adherence alone:

“Sensitization on balanced diet is a key thing here for those who do not know the kinds of foods that they should be taking and when to take them. … Since if you do not eat well and you are taking medication it will not work effectively, the medication works well when you have eaten. And not just any food, every food is important in its own way.” (31 y.o. male, Kenya)

“Sometimes the normal procedure of taking the weights helps to determine whether one is adhering well to the treatment or not, with the trend in the body weights. The clients are given the chance to explain why their body weights are the way they present. Some of the reasons as to why there are low body weights is that various clients have different levels of income. You may be taking the drugs, and there is no good food to maintain the body, and when this one becomes a routine, the drugs will have no effect on the body. It should be that there is some food in the stomach either before or after taking the drugs.” (38 y.o. female, Kenya, beginning virally unsuppressed)

The lay etiology or causal chain for concluding that poor diet leads to high viral load appears to be that food insecurity means people take ART without food, leading to side effects. These side effects then lead to poor adherence, a resultant high viral load, and further weight loss. In parallel, consistent adherence leads one to appear in good health through weight gain. Weight, therefore, stands in as a proxy for viral load in some patients’ perceptions.

Nearly all participants preferred their current location and foresaw adherence challenges if they switched clinics. A young participant suggested that a single provider be assigned to one's case, so the patient could later refer friends to that specific doctor. One provider recognised that clinic transfers are problematic when each clinic has its own system, and recommended electronic medical records (EMR) for all clinics: “*If all facilities would transition from physical files to EMR, then it would be much easier to assist such kinds of transfer-outs because the participant would not need to move with a physical transfer letter*.” (36 y.o. Mobility Coordinator, Kenya)

### Additional services

Many participants faced food insecurity (a driver of mobility) and suggested the clinic provide food supplements such as soy or peanut flour, eggs, beans, rice, ugali, and sugar, or a monthly ration of two tins of flour and clothing. Participants mentioned that prior clinics had done this and sometimes added some money. If the clinic could not give out food supplements, several participants suggested the clinic at least provide refreshments, which, along with transport, would encourage people to come to clinic. As one participant said, “*I think someone can be encouraged to engage more in care when there is some token provided whenever you come to the facility. For example, if there can be transport reimbursement or refreshments, this encourages people to engage more in care*.” (49 y.o. male, Kenya) Given this context, several participants suggested that the clinic provide services to support clients with employment, rent, or money to start a business that doesn't involve hard physical tasks. Finally, one participant also recommended organising interactive community forums where people can share their challenges:

“For those of us who already know their status, we have so many challenges. … Personally, I wish that you would have a forum where you sit with your clients and listen to the challenges that they have and advise them. Allow a general discussion where everyone will give their opinion and the challenges that they are going through. You will find that there are two or three people going through the same thing, and when they are advised together, they will leave that place feeling relieved.” (31 y.o. male, Kenya)

## Discussion

In this qualitative study of a subset of participants from the SEARCH-SAPPHIRE Dynamic Choice Treatment trial, we found participants were pleased with the choice of intervention options tailored for mobile persons that expands on the SEARCH-SAPPHIRE person-centred care model [22], and recommended all options continue and be expanded to others regardless of mobility or HIV status. Home delivery gave participants not only a sense of security should something unexpected befall them but also gave them a sense of being cared for. This personalised care prompted some participants to reconsider their previous spotty ART adherence and make efforts to be more consistent with their ART regimen. Longer refills (up to 6 months) were also appreciated, though the MoH had also implemented three-month-long refills during the pandemic to reduce clinic congestion, overlapping with the SEARCH-SAPPHIRE intervention’s efforts to increase adherence through longer refills [8,42]. Our participants felt that normalising mobility conversations with their providers gave them the confidence to request additional medication outside their regular clinic appointments in case of expected and unexpected travel. Mobility coordinators were useful for the most mobile participants, who could broker discussions around travel plans and strategies to support adherence accordingly.

The travel pack was highly valued for its ability to keep medication (including an extra, “emergency” 14-day supply of ART), money, a travel “checklist” (with reminder prompts about items needed for travel, including ART), and mobility coordinator contact information together in one place while concealing the medication from others and keeping it safe from moisture. It was pre-packed, or easy to pack rapidly, in case of abrupt travel. The travel pack helped overcome several barriers to care engagement and adherence among mobile persons. The surmounted barriers included stigma, because the unmarked wallet or handbag served multiple purposes and did not declare the person’s HIV status; forgetfulness about carrying pills when travelling, since pills and other travel items could be kept in one place; and running out of pills while on the road, due to the additional 14 pills in the travel pack.

The SEARCH-SAPPHIRE Dynamic Choice Treatment approach included elements in common with some DSD models [43], but in addition emphasised a person-centred approach to care delivery, expanding on the original SEARCH-SAPPHIRE person-centred care model [22]. In addition to training that emphasised showing friendliness and care to patients, key elements included adherence counselling and viral load feedback, which have been shown to increase viral suppression [25,35,44–47]. Sharing viral load results with participants may have led to greater engagement not only by increasing ART efficacy beliefs but also because of the greater rapport between patients and providers that came with viral load counselling and the opportunity to co-create adherence strategies [44,48,49]. Such rapport is particularly important with populations whose adherence is ‘precarious’ [44]. For providers, as other researchers have argued, “this one test serves as a unique measure of the coverage, quality, and impact of HIV programmes” [46].

Nonetheless, viral load counselling highlighted certain gaps in health literacy while simultaneously pointing to social aetiologies around what viral load indicates. Findings showed that many participants were confused about the relationship between CD4 count and viral load. Just as people continued previous social observations of AIDS as a ‘slimming disease’ [50], linking viral load and weight, so also they seemed to continue previous understandings about what causes low CD4 counts and apply them to viral load measures (e.g., if stress or poor diet cause low CD4 counts, then these factors will also cause high viral loads). Thus, several participants attributed high viral load to poor diet, which in this region means not fast food, but rather no food; such results are consistent with findings from Malawi [51].

### Limitations

This paper comes with limitations. We did not purposefully select participants who began the study virally unsuppressed. Nor did we stratify our population according to education or ethnicity to see whether these features had any effect on perceptions, attitudes, or outcomes. Our provider data for this paper came only from male mobility coordinators; female mobility coordinators may have been able to offer additional insights. Our data comes from individual interviews, with no focus group discussions to cross-confirm community perceptions. While this may have attenuated some potential social desirability bias, it could not eliminate it entirely. Nonetheless, our participants were forthcoming with recommendations for additional services and improvements to the intervention components.

### Recommendations

Two key recommendations focus on expanding the person-centred approach, first to include elements that were important for (and tailored to) mobile PLHIV specifically, and second, to integrate patient-centred care approaches beyond mobile populations. Regarding the first recommendation, mobility conversations, travel packs, and mobility coordinator-brokered encouragement and counselling have the potential to enhance care for mobile PLHIV by improving communication with providers about the complexity of patient travel planning and needs. In terms of the second recommendation, offering components of the Dynamic Choice Treatment model (originally designed for mobile persons) beyond mobile populations promises to enhance perceptions of quality of care and potential willingness to engage with health care services.

**Fig 1:**
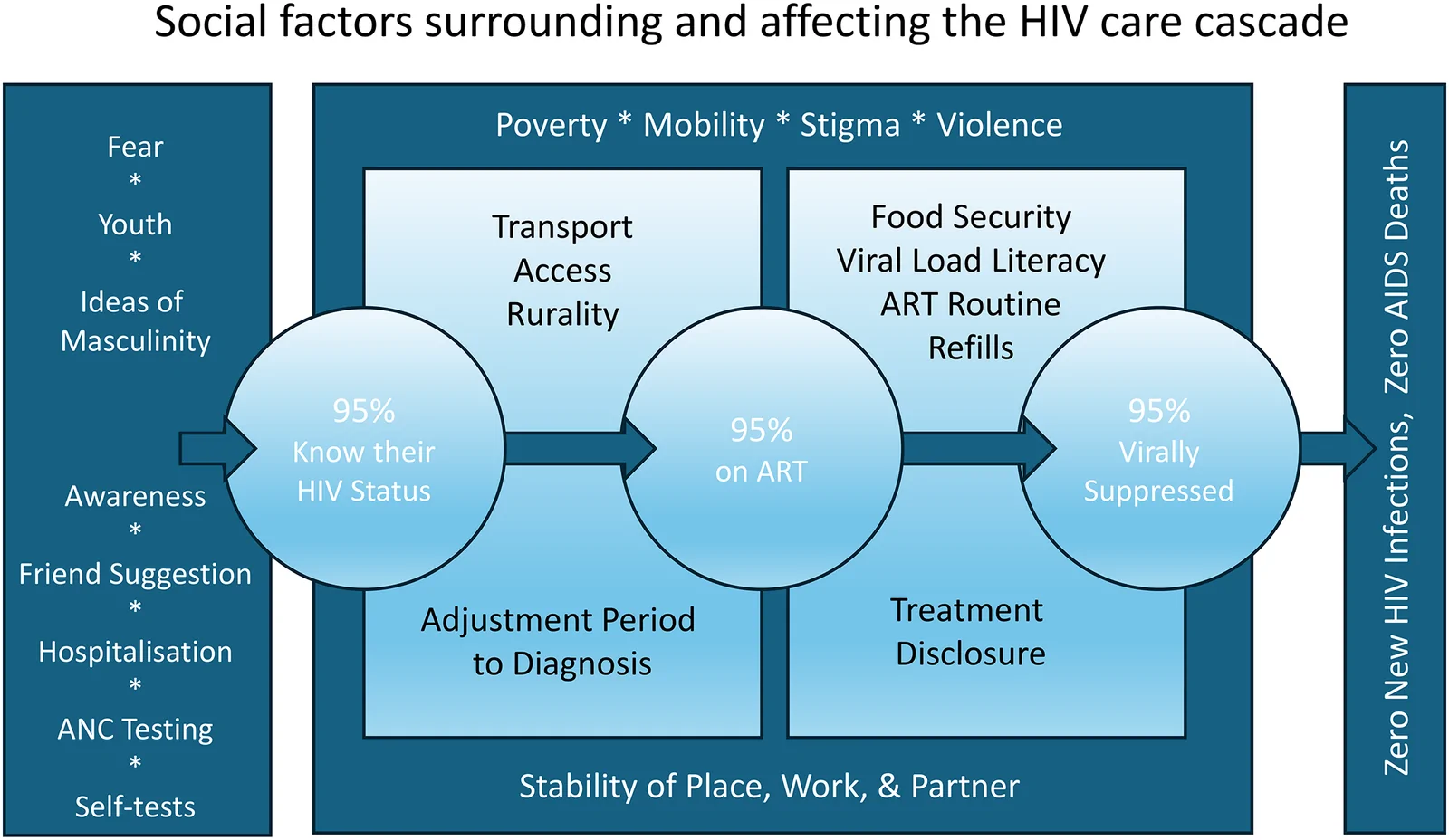
In this figure, the box on the far left represents common pre-existing social factors that facilitate or impede testing for HIV. The middle box contains some common social facilitators and challenges to movement along the cascade of care according to 95-95-95 goals, as described by our participants. The factors at the top tend to relate to impediments, while those in the lower half of the figure relate more to supportive factors. The box at the far right reminds us of our “end game”: zero new HIV infections, zero AIDS deaths.

Local knowledge provided by our participants broadly indicates potentially leverageable opportunities for progress in each of the 95-95-95 goals (see Fig 1) in the areas of food assistance, community forums with providers, and alcohol counselling [52,53]; both participant and provider suggestions for improving care focused on scaling up patient-centred care services to everyone in the community regardless of mobility or HIV status, buttressing services by adding food and employment support to ensure the intervention components achieved their desired ends, and incorporating services to treat other diseases common in the community. In this regard, social assistance, especially in terms of nutritional support for new mothers and their children, may help address a root cause of HIV spread and support biomedical solutions [54]. Food assistance has been shown to generally improve ART adherence in regions of Zambia, Niger, and Mozambique [55]. Counselling on diet can extend beyond ART support to provide an opportunity to emphasise to men and mothers-in-law the importance of nourishing pregnant and post-partum women [56], while viral load counselling with visual aids and culturally appropriate explanations of viral load may help increase local understandings of viral load measurements, with consequent increased viral suppression outcomes [45,57,58]. More generally, the presence of food insecurity also raises questions about how well individual eligibility for DSD models of HIV care, such as minimum body-mass-index, interface with poverty and food insecurity when such poverty alleviation programmes are absent [43]. Person-centred care may bring these variables to light in the clinical encounter and provide an opportunity to refer patients to additional services that can address these needs.

The recommended community forums may allow members to collectively express concerns to providers and give providers an opportunity to reassure the community on a wider scale, while increasing community support for those on treatment, promote healthy couple interactions and familial support for PLHIV, and decreasing the self-stigma that keeps people away from testing and treatment. Community forums might provide a venue for collective solutions for food-insecure members and serve as a means for community members to share information about employment opportunities. Alcohol use has been associated with worse retention in HIV care [59,60] and lower rates of ART initiation (though its relationship with viral suppression for those already initiated on ART has been inconclusive in some studies in the region [61,62]). The participant recommendation to offer alcohol counselling services may also serve to bring new PLHIV into care if offered concurrently with HIV testing or ART initiation [63]. Lastly, providers continue to recommend seamless records coordination between facilities [2]. Ongoing concerns about medication supply chains were also raised, and researchers have drawn attention the need for robust stock management to ensure stable ART distribution [43,64–66].

## Conclusions

A person-centred Dynamic Choice Treatment intervention was popular among this mobile population with HIV and could be expanded to improve care engagement and adherence. Person-centred care fosters the provider and client relationship and includes specific support and flexibility for mobile persons who are unable to engage in care either at structured clinic or community-based visits. Even outside person-centred models of care, the addition of viral load counselling to HIV care delivery is promising because it improves care engagement and adherence, can keep lay health beliefs current with clinical practice by differentiating CD4 and viral load through health literacy counselling, and may ultimately influence viral suppression. Person-centred care models, including those addressing mobile population needs, are therefore acceptable to and appreciated by some mobile populations, and potentially replicable in rural areas where mobility forms a core part of the economic life of the community.

## Supporting information

Table S1 **Codes examined in this study. We deployed a framework analysis drawing on excerpts from the below selected codes to identify emergent themes that answered our primary research questions.** 1. Parent Codes 10 (Mobility) and 11 (Mobility Coordinators) 2. Child Code 9.05 (ART) 3. Parent Code 13 (Adherence) 4. Parent Code 12 (VL Literacy) 5. Services: Parent Code 3 (Clinic Experiences), Child Code 9.01 (Travel Pack), Child Code 9.02 (Longer Refill), Child Code 9.03 (Home based care), Child Code 9.04 (Referrals) 6. Parent Codes 19 (Preferred Location) and 20 (Suggestions).
(DOCX)
