# Supplementary material for: Participant perspectives on a person-centred care model for mobile individuals in HIV care from the SEARCH Dynamic Choice Treatment trial in rural Kenya and Uganda
Source: PLoS One. 2026 Sep 3;21(9):e0356605. doi: 10.1371/journal.pone.0356605 (PMC13541145; doi:10.1371/journal.pone.0356605)
Supplement: Supplementary file 3 [file pone.0356605.s001.docx]

**Table S1. Codes examined in this study.**

***We deployed a framework analysis drawing on excerpts from the below selected codes to identify emergent themes that answered our primary research questions.***

1. Services: Parent Code 3 (Clinic Experiences), Child Code 9.01 (Travel Pack), Child Code 9.02 (Longer Refill), Child Code 9.03 (Home based care), Child Code 9.04 (Referrals)

2. Child Code 9.05 (ART)

3. Parent Codes 10 (Mobility) and 11 (Mobility Coordinators)

4. Parent Code 12 (VL Literacy)

5. Parent Code 13 (Adherence)

6. Parent Codes 19 (Preferred Location) and 20 (Suggestions)
