# Supplementary material for: Participant perspectives on a person-centred care model for mobile individuals in HIV care from the SEARCH Dynamic Choice Treatment trial in rural Kenya and Uganda
Source: PLoS One. 2026 Sep 3;21(9):e0356605. doi: 10.1371/journal.pone.0356605 (PMC13541145; doi:10.1371/journal.pone.0356605)
Supplement: Supplementary file 4 [file pone.0356605.s003.docx]

**Letter of Response to Editors and Reviewers**

**Manuscript ID:**	PONE-D-25-64298

**Manuscript Title:**	Participant perspectives on a person-centred care model for mobile individuals in HIV care from the SEARCH Dynamic Choice Treatment trial in rural Kenya and Uganda

**Dear editors,**

**Thank you for the opportunity to address the helpful comments provided by the reviewers. We have edited the manuscript in response to these comments. We provide both a clean and marked copy of the revised manuscript with this resubmission, and we directly quote the changes in our responses below. We have done our best to undertake a thorough and responsive revision of the manuscript. We hope that the editors and reviewers agree that the revised manuscript is much stronger as a result of these very helpful critiques.**

**Below please find each comment with our response indented and in bold print:**

*Editorial Comments to Author:*

When submitting your revision, we need you to address these additional requirements.

1. Please ensure that your manuscript meets PLOS ONE's style requirements, including those for file naming.

**We have now updated the headings as appropriate.**

2. Please include a complete copy of PLOS’ questionnaire on inclusivity in global research in your revised manuscript. Our policy for research in this area aims to improve transparency in the reporting of research performed outside of researchers’ own country or community. … Please find more information on the policy and a link to download a blank copy of the questionnaire here: [https://journals.plos.org/plosone/s/best-practices-in-research-reporting](https://urldefense.com/v3/__https:/journals.plos.org/plosone/s/best-practices-in-research-reporting__;!!LQC6Cpwp!vNUIDGFM-4o0QHgfPi2b8KT5jM6GR3F0qNKDmPgrZukU-6NgLew0n85IxY6jK7aeyE6eX9_g7TrnRQjDityDxBY$). Please upload a completed version of your questionnaire as Supporting Information when you resubmit your manuscript.

**We have now filled out this questionnaire. Ten of the seventeen co-authors are based in Uganda or Kenya. The co-first author is Kenyan and the co-senior author is Ugandan. The research was thus performed in the countries associated with the majority of the co-authors.**

3. We note that the grant information you provided in the ‘Funding Information’ and ‘Financial Disclosure’ sections do not match. When you resubmit, please ensure that you provide the correct grant numbers for the awards you received for your study in the ‘Funding Information’ section.

**The funding numbers listed below in point 4 are correct.**

4. Thank you for stating the following financial disclosure:  [U.S. National Institute of Allergy and Infectious Diseases (NIAID), the National Heart, Lung, and Blood Institute (NHLBI), and the National Institute of Mental Health (NIMH) and co-funded under award number U01AI150510 (Havlir), with additional support provided by the U.S. National Institutes of Health: grants UH3HD096915 (Havlir), U01AI150510 (Havlir) and K24MH126808 (Camlin). The authors acknowledge sole responsibility for the content of this paper, which does not necessarily represent the official views of the NIH.].

Please state what role the funders took in the study.  If the funders had no role, please state: ""The funders had no role in study design, data collection and analysis, decision to publish, or preparation of the manuscript.""

*Please include this amended Role of Funder statement in your cover letter; we will change the online submission form on your behalf.*

**The funders had no role in study design, data collection and analysis, decision to publish, or preparation of the manuscript.**

5. We note that you have indicated that there are restrictions to data sharing for this study. … Before we proceed with your manuscript, please address the following prompts:

a) If there are ethical or legal restrictions on sharing a de-identified data set, please explain them in detail (e.g., data contain potentially identifying or sensitive patient information, data are owned by a third-party organization, etc.) and who has imposed them (e.g., a Research Ethics Committee or Institutional Review Board, etc.). Please also provide contact information for a data access committee, ethics committee, or other institutional body to which data requests may be sent.

b) If there are no restrictions, please upload the minimal anonymized data set necessary to replicate your study findings to a stable, public repository and provide us with the relevant URLs, DOIs, or accession numbers. Please see [http://www.bmj.com/content/340/bmj.c181.long](https://urldefense.com/v3/__http:/www.bmj.com/content/340/bmj.c181.long__;!!LQC6Cpwp!vNUIDGFM-4o0QHgfPi2b8KT5jM6GR3F0qNKDmPgrZukU-6NgLew0n85IxY6jK7aeyE6eX9_g7TrnRQjDMQOMwMI$) for guidelines on how to de-identify and prepare clinical data for publication. For a list of recommended repositories, please see [https://journals.plos.org/plosone/s/recommended-repositories](https://urldefense.com/v3/__https:/journals.plos.org/plosone/s/recommended-repositories__;!!LQC6Cpwp!vNUIDGFM-4o0QHgfPi2b8KT5jM6GR3F0qNKDmPgrZukU-6NgLew0n85IxY6jK7aeyE6eX9_g7TrnRQjDg4rMWPc$). You also have the option of uploading the data as Supporting Information files, but we would recommend depositing data directly to a data repository if possible.

Please update your Data Availability statement in the submission form accordingly.

**Per prompt a, we reiterate that we do have ethical restrictions on sharing data (as elaborated below in response to point 5). Further, prompt ‘b’ implies that our data is clinical; it is not. Our data is qualitative. We continue to reiterate the same rationale on restricted data sharing (data which may indeed have identifying characteristics) that we have stated to PLoS journals for previously published qualitative work from these studies. We are willing to share but only if the protocols we’ve mentioned in the request for said data are followed.**

5. When completing the data availability statement of the submission form, you indicated that you will make your data available on acceptance. We strongly recommend all authors decide on a data sharing plan before acceptance, as the process can be lengthy and hold up publication timelines. Please note that, though access restrictions are acceptable now, your entire data will need to be made freely accessible if your manuscript is accepted for publication. This policy applies to all data except where public deposition would breach compliance with the protocol approved by your research ethics board. If you are unable to adhere to our open data policy, please kindly revise your statement to explain your reasoning and we will seek the editor's input on an exemption. Please be assured that, once you have provided your new statement, the assessment of your exemption will not hold up the peer review process.

**Again, prompt ‘b’ in item 4 above implies that our data is clinical; it is not. Our data is qualitative, comprised of many in-depth interviews, data which may indeed have identifying characteristics, including HIV status in a region where HIV stigma remains present. We continue to reiterate the same rationale on restricted data sharing that we have stated to PLoS journals for previously published qualitative work from these studies:**

**“Following SEARCH Scientific Review Committee approval of a concept sheet summarizing the analyses to be done, de-identified study data and a data dictionary may be made available in a secure online repository with a signed data access agreement. Further inquiries can be directed to the SEARCH Scientific Review Committee via** [**Douglas.Black@ucsf.edu**](mailto:Douglas.Black@ucsf.edu)**.”**

6. Please include captions for your Supporting Information files at the end of your manuscript, and update any in-text citations to match accordingly. Please see our Supporting Information guidelines for more information: [http://journals.plos.org/plosone/s/supporting-information](https://urldefense.com/v3/__http:/track.editorialmanager.com/CL0/http:*2F*2Fjournals.plos.org*2Fplosone*2Fs*2Fsupporting-information/1/010f019dd7311902-4dcebdd9-4e72-4701-b46e-888b8221f152-000000/_tThcPskuaTbGLEgnbQCCYyCnysfaps9EnOXhZzFCk8=258__;JSUlJSU!!LQC6Cpwp!vNUIDGFM-4o0QHgfPi2b8KT5jM6GR3F0qNKDmPgrZukU-6NgLew0n85IxY6jK7aeyE6eX9_g7TrnRQjD4mgXI60$).

**We only had a figure 1, whose caption was included in the manuscript already. We have now duplicated that caption following the references.**

**

*Reviewer 1 Comments to the Author*

**

Overall, the writing is acceptable. The manuscript adheres to acceptable technical standards. The data support the conclusions.

The title needs to be revised as it doesn't quite reflect the content. Perhaps "Perceptions and feelings of participants..." would be preferable to "Perspectives of participants..."

**Thank you for this suggestion. Respectfully, we chose ‘Participant perspectives’ to include both patient/client feelings (part one of the results) and recommendations from both patients and providers (part two of the results). Changing the title to perceptions and feelings would not cover the second part of the results, and may give the impression we are only reporting patient responses, rather than both patient and provider responses.**

Explain what you mean in Table 1, specifically the row concerning arm and its control and intervention modalities.

**Thank you for suggesting we clarify this. We have now moved a paragraph from the background to the methods section to be more explicit about these two arms.**

Explain Table S1 more clearly and provide relevant details to enhance understanding of the methodology and facilitate comprehension for readers.

**Thank you for noting this. We have now added the following to Table S1: “We deployed a framework analysis drawing on excerpts from the following selected codes to identify emergent themes that answered our primary research questions.”**

**We earlier described the method as follows: “An eight-person team (LO, AO, CA, FA, IM, TA, JL, JJP) then coded the transcripts with this codebook. Following coding, we deployed a framework analysis of selected codes to identify emergent themes that answered our primary research questions. We exported coded excerpts from the following broad codes: mobility, ART and adherence, viral load literacy, service perceptions, and recommendations. (The specific codes are in Table S1.) These excerpts included data from both patient-client and provider participants.”**

**We have also added citations to sources describing framework analysis in more detail: Furber 2010, Gale 2013, Parkinson 2016, and Goldsmith 2021.**

Review the numbering of titles and subtitles in the results section (for example, 1.0, 1.1, and directly 1.2.1, among others). This inconsistency makes it difficult to follow the chronological order of your results. The same applies to line 258.

**We have now added -.0 as appropriate. We have also changed the heading style (from body to heading 1, 2, etc) per editorial request.**

For the discussion, a more thorough restructuring will be necessary, and I suggest starting with the limitations of your study (lines 498 to 505 should be placed first).

**Thank you for this suggestion. Placing limitations first is not standard format for academic papers of this type. We have therefore kept the limitations subsection towards the end of the discussion and before the recommendations.**

Also, create a section on ethical considerations and ethical approval immediately following the methodology.

**Thank you for this suggestion. The ethics paragraph was included in the title page submitted to the editors, who requested it only appear once (on the title page, not in the manuscript). For reference, the section reads as follows:**

**“The University of California San Francisco Committee on Human Research, the Ethical Review Committee of the Kenya Medical Research Institute, the Makerere University School of Medicine Research and Ethics Committee and the Uganda National Council for Science and Technology all approved this study (UCSF 20-32144; KEMRI No. 4173; UNCST No. HS1239ES; SOMREC No. 2020-29). All study participants provided written and informed consent to participate in interviews.”**

**We also have an author position statement in the title page, which again, likely does not appear in the anonymized manuscript portion: “*Author(s) position statement(s).* The team-based analytical approach was grounded in the cultural experience of research team authors who had first-hand understanding of the study setting (AO, CA, FA, LO, TOA). Further, the same research team members who gathered data in Dholuo (LO, AO, TAO), Runyankole (CA, FA), and Swahili (AO), participated in data analysis and confirmation of the findings to which other team members contributed (JJP, CSC). This ensured cultural rigour so that data interpretation respectfully captured the nuances of participants’ voices. Additionally, since we have the linguistic capacity (JJP), we also have a team commitment to cite at least one source from Francophone Africa and one from Lusophone Africa per article, in order to surmount colonially-rooted academic barriers between nations and regions in Africa. Despite these provisions, we recognise the possibility of contested realities and the relativity of our analysis.”**

**

*Reviewer 2 Comments to the Author*

**

Thank you for looking this important topic of great interest.
Please find my comments in the attached documments and below:

Ln 7 :Better specify the number of months or range of months.

**We agree, and have now specified “six-month” refills in the abstract.**

Ln31:" Suppressed"

**Thank you for catching this important typo.**

Ln:54-66:Patient-Centred care is an holistic care model in consultation with well defined elements : 1.Exploring both disease and illness experiences . 2. Understanding the whole person. 3. Finding the common ground. 4 Enhancing pateint-Doctor relationship. 5 Incorporating prevention and health care promotion. 6. Being realistic. Preferences come under understanding the whole person including their context. Please look into these components of the Patient-Centered care and rephrase this section in a way to remove adjectival impressions and foster your primary intentions of PCC on mobile PWHIV .

**Thank you for this list. While we agree with the six domains you’ve listed (and we wish you’d provided a citation that we could have incorporated into the paper), our study was embedded in a larger trial. In this paper, we cited the references to person-centred care used in the main study to maintain continuity with the design of the larger trial and to align this paper with those from the larger trial.**

**Additionally, we believe we had already addressed five of the six domains you listed (the exception being disease/illness experiences, which was not the focus of this paper) as follows:**

***Understanding the whole person*: “patient-centred care can reach all populations because it is concerned with addressing patients’ needs for care that affirms their agency and dignity and is responsive to their cultural values, understanding the whole person.” (We have now added the final phrase.)**

***Finding common ground*: “Such relationships are fostered through empathy and communication skills sensitive to the life stage or circumstances of the patient.”**

***Enhancing the patient-provider relationship*: “Additional components of patient-centred care include friendly providers who cultivate positive patient-provider and patient-clinic relationships and reduce stigma, distinguish between those who need intensive counselling and those who can engage with streamlined care, and enhance knowledge of HIV and ART as necessary.”**

***Including health literacy promotion*: “These approaches that establish rapport, broach sensitive topics, or engage in motivational interviewing can help promote greater engagement in patients’ own healthcare, while potentially decreasing physician burnout.”**

***Being realistic*: “Patient-centred care recognises the heterogeneity of patients in order to apprise individuals accurately about their levels of risk, while working with them to find the most appropriate means to address their current circumstances.”**

Ln83 :replace with "and to"

**Adding ‘and’ will change our intended meaning, which is “in order to”. We have therefore specified, “in order to”.**

Ln84-86:Reconstitute this as a separate statement please.

**We have now broken the longer sentence into two separate sentences as follows: “This paper therefore seeks to describe participant and provider perceptions of the intervention in order to identify what may have led to higher patient engagement in care while also presenting participant recommendations for improvement of individual components of that model. Through these approaches, we sought to uncover how much of the intervention is potentially scalable or replicable for health systems in other rural areas where mobility forms a core part of the economic life of the community.”**

Ln88:This "Method" contains useful information to understand the purpose of your study and context but, unfortunately, still not optimal in research requirement.
1. The inclusion/exclusion criteria of your purposive /judgmental samples is very important.
2. Is disease prevalence the only factor in deciding the clinics from which the participants were extracted?

**Both Reviewer 1 and Reviewer 2 have noted similar questions here, and we are grateful they have brought this to our attention. We have now moved a paragraph from the background to the methods section to be more explicit about these two arms and ensure readers recognise the qualitative substudy was part of the main study. The moved paragraph is as follows:**

**“Intervention components have been described in more detail in the primary paper (8). Briefly, its seven components included provision of a “travel pack” containing a travel checklist (with mobility coordinator phone number) to remind participants of what to pack when travelling, discreet ART packing options, and an emergency supply of 14 extra ART pills; a mobility coordinator who would ask participants about their travel plans at each visit and coordinate care with them accordingly; “hotline” phone and SMS access to reach the mobility coordinator as needed; extended refills up to 6 months; community-based delivery of medication for persons unable to return to clinic; assistance in navigating out-of-community ART refills; and transfers of care to out-of-community HIV clinics (when out-migrating without a plan to return) (8). Viral load counselling for participants was also a core component of the SEARCH-SAPPHIRE patient-centred model (22, 35). Control arm participants received usual care and follow-up delivered by the Ministries of Health in the respective countries.”**

**Further, we specified the purposive sampling categories: “…purposively selected participants who received the Dynamic Choice Treatment intervention. The selection was balanced by sex, livelihood or profession, and community”. The suggestion seems to elide the selection of communities for the main trial – which we are not discussing in this paper, and whose results have already been published – with those participants drawn from the main study, and who are the focus of this paper. In terms of the main study, yes, it was “conducted in 5 rural clinics in western Kenya and southwestern Uganda with high HIV prevalence according to Ministry of Health (MoH) data” AND “the Dynamic Choice Treatment study was one of several trials in the first phase of the study. … the SEARCH Collaboration designed a differentiated, patient-centred clinical care model intervention to address the needs of mobile participants with HIV” as one of those trials. The other trials included dynamic choice prevention at antenatal clinics, outpatient clinics, and community settings. Those results have also been published elsewhere.**

Ln91:Follow this by mentioning the sectoral involvements e.g : "multisectoral stategy of HIV Medical officers and psychologist to reach...."

**Thank you for suggesting we clarify what ‘multisectoral’ means in this context. We have now specified this as “the SEARCH-SAPPHIRE Study (NCT04810650) deploys a multisectoral strategy drawn from implementation science, precision medicine, and population health to reach “persistent driver” populations.”** [**https://www.searchendaids.com/**](https://www.searchendaids.com/)

Table 1: I am unclear about this section and it is inconsistent with what you have been discussing so far .
Do you mean that some participants do not receive the treatment?
Are you doing a clinical trial or what ?

**Thank you for asking clarification. We did a community intervention study. Control participants received usual MoH care. We have now revised the methods section to make this more explicit.**

Ln140:Please keep to a particular form of incorporating the verbatim quotes.
I prefer a complete separation from the lead-in paragraph because of the grammatical errors that might be in the verbatim quotes.
Kindly correct this

**Thank you for this observation. Our particular form is to separate and indent quotes that are longer than three lines, per reviewer requests and standard format for qualitative and social science research papers. Quotes that are shorter than that remain within the framing paragraph.**

Ln184-185: Isolate this as participant's response and start another paragraph/ statement after that

**We have now started a new paragraph after this participant quote. We have kept the quote embedded in the prior paragraph per our quote citation style, following standard qualitative and social science paper format.**

Ln195-196:Replace with " Overall, Patient-Clients liked being asked about their mobility plans because it gave them a chance to work with the provider and strategise support for ART adherence as expressed by similar views from different participants from two different sites":

**We have adopted a version of the above wording and broken out the short quote into its own paragraph because of the resultant framing.**

Ln219-220:Replace with " Knowledge gap was identified between CD4 count and Viral load as expressed by a participant and a corrective information by a mobility coordinator" :

**Thank you for this suggested wording. However, the issue in question wasn’t simply a knowledge gap. Participants expressed outright confusion between the CD4 and VL measures. Adopting the suggested language will also eliminate our point that some patients thought high CD4 and high VL were both desirable.**

Ln238-240:Replace with : Having adequate knowledge about Viral load, adherence and health outcomes was reported to have a positive motivating impacts on the treatment compliance of the participants.

**In this instance, we have opted to keep the prior wording, as the suggested revision is in passive voice. Maintaining active voice highlights the agency of our participants.**

Ln252:put :that

**We have added the relative pronoun to introduce the subordinate clause.**

Ln253:put:Participants

**We have added the noun, necessitating a change from a substantive to an adjective.**

Ln253:as shown in the context of.....

**We respectfully disagree with this suggestion, as it will change the meaning of our sentence(s) and distort what we intend to say.**

Ln258-260: The three to six month chronic medication refill time introduced during the Covid 19 pandemic reduced the in-facility congestion, but the longer refill time introduced in this study achieved other additional benefits ranging from .......

**We are unclear on what needs to be done here. The control arm participants received usual MoH care, while the intervention arm participants received longer refills by study design. The suggested revision eliminates this difference between the arms.**

Ln269-270:The stigma associated with recurrent clinic visits as HIV patient has been reduced by a longer refill time introduced in this study

**We agree that making stigma explicit is important here. We have therefore added a variant of the above wording as follows: “Longer refills meant fewer clinic appointments and less community speculation about why some participants were ‘always’ at the facility, allowing participants to avoid the stigma associated with recurrent clinic visits.”**

Ln327-328: What exactly are you trying to say here?

**Thank you for pointing this out. We neglected to specify the ‘requests’ in ‘streamline transfer letter requests’.**

Ln357:This terms are reserved for experimental studies or a mixed method research in which probably there may be an interview of participants in each of the groups.

**We conducted a community-cluster intervention trial, and thus we had both a control and an intervention arm. We have now clarified this in our revision to the results.**

Ln364: Providers were satisfied that six-month refill time and telephonic conversation with the participants were valuable in their care :

**Again, this suggestion changes the focus of the quote and our intentions for including it.**

Ln374-376:How is this verbatim quote relevant to mobility conversation?

**As the participant says, he finds these conversations with the provider motivating – they encourage him to continue to adhere to clinic appointments.**

Ln378-379:Do you want to say that both men and women's packs were popular ?

**We mean to say that the men’s packs were popular with the women – they tended to prefer men’s packs to women’s packs – which is what we have written.**

Ln440: This could be in form of monetary assistance for small businesses or tokens to augment their low earnings.

**The suggested change is not what the participants said. We have retained the original language.**

****

**Again, we appreciate the helpful comments provided by the reviewers and editor. Thank you for the opportunity to have our manuscript considered for publication in PLoS One.**

**Sincerely,**

**Jason Johnson-Peretz**
