# Supplementary material for: Participant perspectives on a person-centred care model for mobile individuals in HIV care from the SEARCH Dynamic Choice Treatment trial in rural Kenya and Uganda
Source: PLoS One. 2026 Sep 3;21(9):e0356605. doi: 10.1371/journal.pone.0356605 (PMC13541145; doi:10.1371/journal.pone.0356605)
Supplement: Supplementary file 5 [file pone.0356605.s005.docx]

**Letter of Response to Editors and Reviewers**

**Manuscript ID:**	PONE-D-25-64298

**Manuscript Title:**	Participant perspectives on a person-centred care model for mobile individuals in HIV care from the SEARCH Dynamic Choice Treatment trial in rural Kenya and Uganda

**Dear editors,**

**Thank you for the opportunity to clarify the additional comments provided by the reviewers. We request editorial decision on our responses.**

**Below please find each comment with our response indented and in bold print:**

**

*Reviewer 1 Comments to the Author*

**

Thank you for the clarifications provided.

A few comments regarding the answers provided and to improve the quality of your work:
1. I was unable to access the supplementary table S1 (line 146) (which is part of the additional information to be provided in the appendix).

**Thank you for bringing this to the editor’s attention. I am on the other side of the platform’s interface, and thus am uncertain how to help.**

2. Lines 3-4: We sought to explore participant perceptions of a patient-centered model aimed at enhancing care engagement through ‘dynamic choice’ treatment services for mobile individuals.

What distinction do you make between "Participant perspectives" (used in the title of your research) and "Participant perceptions" (used here in line 3)?
I believe the suggestion I made to you addresses this point and emphasizes the importance of maintaining the parallel structure and the appropriate word choice. I note a fundamental difference between perceptions and perspectives, unless I am mistaken.

**Thank you for noting this. Because we are not presenting a phenomenological account of participants’ experiences, we have use the two terms synonymously, focusing on the overlap in semantic range shared by the two terms.**

3. Lines 104-105: I think It is necessary and preferable to provide details within this manuscript, rather than referencing another article, to ensure precision and clarity. This would be the ideal time to fully explain the type of your study and its specific characteristics.

**Thank you for noting this. In the revised manuscript we submitted, we did elaborate on those components of the main study essential to understanding the qualitative component, as follows: “Briefly, its seven components included provision of a “travel pack” containing a travel checklist (with mobility coordinator phone number) to remind participants of what to pack when travelling, discreet ART packing options, and an emergency supply of 14 extra ART pills; a mobility coordinator who would ask participants about their travel plans at each visit and coordinate care with them accordingly; “hotline” phone and SMS access to reach the mobility coordinator as needed; extended refills up to 6 months; community-based delivery of medication for persons unable to return to clinic; assistance in navigating out-of-community ART refills; and transfers of care to out-of-community HIV clinics (when out-migrating without a plan to return) (8). Viral load counselling for participants was also a core component of the SEARCH-SAPPHIRE patient-centred model (22, 35). Control arm participants received usual care and follow-up delivered by the Ministries of Health in the respective countries.”**

**Additionally, in the methods section we earlier indicated what sort of trial the main study was: “the SEARCH Collaboration designed a differentiated, patient-centred clinical care model intervention to address the needs of mobile participants with HIV.”**

Best regards, and may you find the observations helpful.

**Thank you for your close read of the manuscript. Your observations have overall improved the paper.**

**

*Reviewer 2 Comments to the Author*

**

Thank you for this very important study of great importance and relevance , especially, in sub-saharan Africa. You have responded to my concerns appropriately .

Simple observation and comment about N and n as in the manuscript attached .
Review this in the Abstract, tables and wherever applicable in the manuscript.

**Thank you for this note. Because this is a qualitative paper using a subset of the main trial’s number of participants (where quantitative differentiation between N and n is vital for clariy), we have chosen to use N for each separate sample of the qualitative study. We justify this because the two samples in the qualitative study are differentiated by those who *received* the study components (or control care) and those who *delivered* it. We feel that using N for both would imply that the providers also enrolled in the main trial as participants. We have therefore kept majuscule N for the purposes of this paper.**

****

**Again, we appreciate the helpful comments provided by the reviewers and editor. Thank you for the opportunity to have our manuscript considered for publication in PLoS One.**
