# Supplementary material for: Participant perspectives on a person-centred care model for mobile individuals in HIV care from the SEARCH Dynamic Choice Treatment trial in rural Kenya and Uganda
Source: PLoS One. 2026 Sep 3;21(9):e0356605. doi: 10.1371/journal.pone.0356605 (PMC13541145; doi:10.1371/journal.pone.0356605)
Supplement: Peer review history

# Peer review: Decision Letter 0

**DOI:** 10.1371/journal.pone.0356605.r001

**Authors:** Richard Makurumidze

28 Apr 2026

Dear Dr. Johnson-Peretz,

Please submit your revised manuscript by Jun 12 2026 11:59PM. If you will need more time than this to complete your revisions, please reply to this message or contact the journal office at plosone@plos.org. When you're ready to submit your revision, log on to https://www.editorialmanager.com/pone/ and select the 'Submissions Needing Revision' folder to locate your manuscript file.

- A letter that responds to each point raised by the academic editor and reviewer(s). You should upload this letter as a separate file labeled 'Response to Reviewers'.
- A marked-up copy of your manuscript that highlights changes made to the original version. You should upload this as a separate file labeled 'Revised Manuscript with Track Changes'.
- An unmarked version of your revised paper without tracked changes. You should upload this as a separate file labeled 'Manuscript'.

If you would like to make changes to your financial disclosure, please include your updated statement in your cover letter. Guidelines for resubmitting your figure files are available below the reviewer comments at the end of this letter.

If applicable, we recommend that you deposit your laboratory protocols in protocols.io to enhance the reproducibility of your results. Protocols.io assigns your protocol its own identifier (DOI) so that it can be cited independently in the future. For instructions see: https://journals.plos.org/plosone/s/submission-guidelines#loc-laboratory-protocols. Additionally, PLOS ONE offers an option for publishing peer-reviewed Lab Protocol articles, which describe protocols hosted on protocols.io. Read more information on sharing protocols at https://plos.org/protocols?utm_medium=editorial-email&utm_source=authorletters&utm_campaign=protocols.

As the corresponding author, your ORCID iD is verified in the submission system and will appear in the published article. PLOS supports the use of ORCID, and we encourage all coauthors to register for an ORCID iD and use it as well. Please encourage your coauthors to verify their ORCID iD within the submission system before final acceptance, as unverified ORCID iDs will not appear in the published article. *Only*  the individual author can complete the verification step; PLOS staff *cannot*  verify ORCID iDs on behalf of authors.

We look forward to receiving your revised manuscript.

Kind regards,

Richard Makurumidze

Academic Editor

PLOS One

Journal Requirements:

When submitting your revision, we need you to address these additional requirements.

1. Please ensure that your manuscript meets PLOS ONE's style requirements, including those for file naming. The PLOS ONE style templates can be found at

https://journals.plos.org/plosone/s/file?id=wjVg/PLOSOne_formatting_sample_main_body.pdf and

https://journals.plos.org/plosone/s/file?id=ba62/PLOSOne_formatting_sample_title_authors_affiliations.pdf

2. Please include a complete copy of PLOS’ questionnaire on inclusivity in global research in your revised manuscript. Our policy for research in this area aims to improve transparency in the reporting of research performed outside of researchers’ own country or community. The policy applies to researchers who have travelled to a different country to conduct research, research with Indigenous populations or their lands, and research on cultural artefacts. The questionnaire can also be requested at the journal’s discretion for any other submissions, even if these conditions are not met. Please find more information on the policy and a link to download a blank copy of the questionnaire here: https://journals.plos.org/plosone/s/best-practices-in-research-reporting. Please upload a completed version of your questionnaire as Supporting Information when you resubmit your manuscript.

3. We note that the grant information you provided in the ‘Funding Information’ and ‘Financial Disclosure’ sections do not match.

When you resubmit, please ensure that you provide the correct grant numbers for the awards you received for your study in the ‘Funding Information’ section.

4. Thank you for stating the following financial disclosure:  [U.S. National Institute of Allergy and Infectious Diseases (NIAID), the National Heart, Lung, and Blood Institute (NHLBI), and the National Institute of Mental Health (NIMH) and co-funded under award number U01AI150510 (Havlir), with additional support provided by the U.S. National Institutes of Health: grants UH3HD096915 (Havlir), U01AI150510 (Havlir) and K24MH126808 (Camlin). The authors acknowledge sole responsibility for the content of this paper, which does not necessarily represent the official views of the NIH.].

Please state what role the funders took in the study.  If the funders had no role, please state: ""The funders had no role in study design, data collection and analysis, decision to publish, or preparation of the manuscript.""

If this statement is not correct you must amend it as needed.

Please include this amended Role of Funder statement in your cover letter; we will change the online submission form on your behalf.

5. We note that you have indicated that there are restrictions to data sharing for this study. For studies involving human research participant data or other sensitive data, we encourage authors to share de-identified or anonymized data. However, when data cannot be publicly shared for ethical reasons, we allow authors to make their data sets available upon request. For information on unacceptable data access restrictions, please see http://journals.plos.org/plosone/s/data-availability#loc-unacceptable-data-access-restrictions.

Before we proceed with your manuscript, please address the following prompts:

a) If there are ethical or legal restrictions on sharing a de-identified data set, please explain them in detail (e.g., data contain potentially identifying or sensitive patient information, data are owned by a third-party organization, etc.) and who has imposed them (e.g., a Research Ethics Committee or Institutional Review Board, etc.). Please also provide contact information for a data access committee, ethics committee, or other institutional body to which data requests may be sent.

b) If there are no restrictions, please upload the minimal anonymized data set necessary to replicate your study findings to a stable, public repository and provide us with the relevant URLs, DOIs, or accession numbers. Please see http://www.bmj.com/content/340/bmj.c181.long for guidelines on how to de-identify and prepare clinical data for publication. For a list of recommended repositories, please see https://journals.plos.org/plosone/s/recommended-repositories. You also have the option of uploading the data as Supporting Information files, but we would recommend depositing data directly to a data repository if possible.

Please update your Data Availability statement in the submission form accordingly.

5. When completing the data availability statement of the submission form, you indicated that you will make your data available on acceptance. We strongly recommend all authors decide on a data sharing plan before acceptance, as the process can be lengthy and hold up publication timelines. Please note that, though access restrictions are acceptable now, your entire data will need to be made freely accessible if your manuscript is accepted for publication. This policy applies to all data except where public deposition would breach compliance with the protocol approved by your research ethics board. If you are unable to adhere to our open data policy, please kindly revise your statement to explain your reasoning and we will seek the editor's input on an exemption. Please be assured that, once you have provided your new statement, the assessment of your exemption will not hold up the peer review process.

6. Please include captions for your Supporting Information files at the end of your manuscript, and update any in-text citations to match accordingly. Please see our Supporting Information guidelines for more information: http://journals.plos.org/plosone/s/supporting-information.

7. If the reviewer comments include a recommendation to cite specific previously published works, please review and evaluate these publications to determine whether they are relevant and should be cited. There is no requirement to cite these works unless the editor has indicated otherwise.

[Note: HTML markup is below. Please do not edit.]

Reviewers' comments:

Reviewer's Responses to Questions

**Comments to the Author**

1. Is the manuscript technically sound, and do the data support the conclusions?

Reviewer #1: Yes

Reviewer #2: Partly

**********

2. Has the statistical analysis been performed appropriately and rigorously?

Reviewer #1: Yes

Reviewer #2: N/A

**********

3. Have the authors made all data underlying the findings in their manuscript fully available?

The PLOS Data policy

Reviewer #1: Yes

Reviewer #2: Yes

**********

4. Is the manuscript presented in an intelligible fashion and written in standard English?

Reviewer #1: Yes

Reviewer #2: No

**********

Reviewer #1: Overall, the writing is acceptable. The manuscript adheres to acceptable technical standards. The data support the conclusions.

The title needs to be revised as it doesn't quite reflect the content. Perhaps "Perceptions and feelings of participants..." would be preferable to "Perspectives of participants..."

Explain what you mean in Table 1, specifically the row concerning arm and its control and intervention modalities.

Explain Table S1 more clearly and provide relevant details to enhance understanding of the methodology and facilitate comprehension for readers.

Review the numbering of titles and subtitles in the results section (for example, 1.0, 1.1, and directly 1.2.1, among others). This inconsistency makes it difficult to follow the chronological order of your results. The same applies to line 258.

For the discussion, a more thorough restructuring will be necessary, and I suggest starting with the limitations of your study (lines 498 to 505 should be placed first).

Also, create a section on ethical considerations and ethical approval immediately following the methodology.

Reviewer #2: Dear Author,

Thank you for looking this important topic of great interest.

Please find my comments in the attached documments and below:

Ln 7 :Better specify the number of months or range of months.

Ln31:" Suppressed"

Ln:54-66:Patient-Centred care is an holistic care model in consultation with well defined elements : 1.Exploring both disease and illness experiences . 2. Understanding the whole person. 3. Finding the common ground. 4 Enhancing pateint-Doctor relationship. 5 Incorporating prevention and health care promotion. 6. Being realistic.

Preferences come under understanding the whole person including their context.

Please look into these components of the Patient-Centered care and rephrase this section in a way to remove adjectival impressions and foster your primary intentions of PCC on mobile PWHIV .

Ln83 :replace with "and to"

Ln84-86:Reconstitute this as a separate statement please.

Ln88:This "Method" contains useful information to understand the purpose of your study and context but, unfortunately, still not optimal in research requirement.

1. The inclusion/exclusion criteria of your purposive /judgmental samples is very important.

2. Is disease prevalence the only factor in deciding the clinics from which the participants were extracted?

Ln91:Follow this by mentioning the sectoral involvements e.g : "multisectoral stategy of HIV Medical officers and psychologist to reach...."

Table 1: I am unclear about this section and it is inconsistent with what you have been discussing so far .

Do you mean that some participants do not receive the treatment?

Are you doing a clinical trial or what ?

Ln140:Please keep to a particular form of incorporating the verbatim quotes.

I prefer a complete separation from the lead-in paragraph because of the grammatical errors that might be in the verbatim quotes.

Kindly correct this

Ln184-185: Isolate this as participant's response and start another paragraph/ statement after that

Ln195-196:Replace with " Overall, Patient-Clients liked being asked about their mobility plans because it gave them a chance to work with the provider and strategise support for ART adherence as expressed by similar views from different participants from two different sites":

Ln219-220:Replace with " Knowledge gap was identified between CD4 count and Viral load as expressed by a participant and a corrective information by a mobility coordinator" :

Ln238-240:Replace with : Having adequate knowledge about Viral load, adherence and health outcomes was reported to have a positive motivating impacts on the treatment compliance of the participants.

Ln252:put :that

Ln253:put:Participants

Ln253:as shown in the context of.....

Ln258-260: The three to six month chronic medication refill time introduced during the Covid 19 pandemic reduced the in-facility congestion, but the longer refill time introduced in this study achieved other additional benefits ranging from .......

Ln269-270:The stigma associated with recurrent clinic visits as HIV patient has been reduced by a longer refill time introduced in this study

Ln327-328: What exactly are you trying to say here?

Ln357:This terms are reserved for experimental studies or a mixed method research in which probably there may be an interview of participants in each of the groups.

Ln364: Providers were satisfied that six-month refill time and telephonic conversation with the participants were valuable in their care :

Ln374-376:How is this verbatim quote relevant to mobility conversation?

Ln378-379:Do you want to say that both men and women's packs were popular ?

Ln440: This could be in form of monetary assistance for small businesses or tokens to augment their low earnings.

**********

what does this mean?). If published, this will include your full peer review and any attached files.

If you choose “no”, your identity will remain anonymous but your review may still be made public.

**Do you want your identity to be public for this peer review?**  For information about this choice, including consent withdrawal, please see our Privacy Policy

Reviewer #1: **Yes:** KETEHOUNDJE Désiré

Reviewer #2: **Yes:** Adeloye Amoo Adeniji (MBBS; MMed; FCFP; FACRRM)

**********

[NOTE: If reviewer comments were submitted as an attachment file, they will be attached to this email and accessible via the submission site. Please log into your account, locate the manuscript record, and check for the action link "View Attachments". If this link does not appear, there are no attachment files.]

To ensure your figures meet our technical requirements, please review our figure guidelines: https://journals.plos.org/plosone/s/figures

You may also use PLOS’s free figure tool, NAAS, to help you prepare publication quality figures: https://journals.plos.org/plosone/s/figures#loc-tools-for-figure-preparation.

NAAS will assess whether your figures meet our technical requirements by comparing each figure against our figure specifications.

Attachment Submitted filename: PONE-D-25-64298.pdf

# Author comment: Author response to Decision Letter 1

**DOI:** 10.1371/journal.pone.0356605.r002

11 May 2026

Please see uploaded letter.

Attachment Submitted filename: DCT Main Qual Paper Response to Reviewers 2026-04-29.docx

# Peer review: Decision Letter 1

**DOI:** 10.1371/journal.pone.0356605.r003

**Authors:** Richard Makurumidze

11 Jun 2026

Dear Dr. Johnson-Peretz,

Thank you for submitting your manuscript to PLOS ONE. After careful consideration, we feel that it has merit but does not fully meet PLOS ONE’s publication criteria as it currently stands. Therefore, we invite you to submit a revised version of the manuscript that addresses the points raised during the review process.

Please submit your revised manuscript by Jul 26 2026 11:59PM. If you will need more time than this to complete your revisions, please reply to this message or contact the journal office at plosone@plos.org. When you're ready to submit your revision, log on to https://www.editorialmanager.com/pone/ and select the 'Submissions Needing Revision' folder to locate your manuscript file.

- A letter that responds to each point raised by the academic editor and reviewer(s). You should upload this letter as a separate file labeled 'Response to Reviewers'.
- A marked-up copy of your manuscript that highlights changes made to the original version. You should upload this as a separate file labeled 'Revised Manuscript with Track Changes'.
- An unmarked version of your revised paper without tracked changes. You should upload this as a separate file labeled 'Manuscript'.

If you would like to make changes to your financial disclosure, please include your updated statement in your cover letter. Guidelines for resubmitting your figure files are available below the reviewer comments at the end of this letter.

If applicable, we recommend that you deposit your laboratory protocols in protocols.io to enhance the reproducibility of your results. Protocols.io assigns your protocol its own identifier (DOI) so that it can be cited independently in the future. For instructions see: https://journals.plos.org/plosone/s/submission-guidelines#loc-laboratory-protocols. Additionally, PLOS ONE offers an option for publishing peer-reviewed Lab Protocol articles, which describe protocols hosted on protocols.io. Read more information on sharing protocols at https://plos.org/protocols?utm_medium=editorial-email&utm_source=authorletters&utm_campaign=protocols.

As the corresponding author, your ORCID iD is verified in the submission system and will appear in the published article. PLOS supports the use of ORCID, and we encourage all coauthors to register for an ORCID iD and use it as well. Please encourage your coauthors to verify their ORCID iD within the submission system before final acceptance, as unverified ORCID iDs will not appear in the published article. *Only*  the individual author can complete the verification step; PLOS staff *cannot*  verify ORCID iDs on behalf of authors.

We look forward to receiving your revised manuscript.

Kind regards,

Richard Makurumidze

Academic Editor

PLOS One

Journal Requirements:

If the reviewer comments include a recommendation to cite specific previously published works, please review and evaluate these publications to determine whether they are relevant and should be cited. There is no requirement to cite these works unless the editor has indicated otherwise.

Please review your reference list to ensure that it is complete and correct. If you have cited papers that have been retracted, please include the rationale for doing so in the manuscript text, or remove these references and replace them with relevant current references. Any changes to the reference list should be mentioned in the rebuttal letter that accompanies your revised manuscript. If you need to cite a retracted article, indicate the article’s retracted status in the References list and also include a citation and full reference for the retraction notice.

[Note: HTML markup is below. Please do not edit.]

Reviewers' comments:

Reviewer's Responses to Questions

**Comments to the Author**

Reviewer #1: (No Response)

Reviewer #2: All comments have been addressed

**********

2. Is the manuscript technically sound, and do the data support the conclusions?

Reviewer #1: Partly

Reviewer #2: Yes

**********

3. Has the statistical analysis been performed appropriately and rigorously?

Reviewer #1: N/A

Reviewer #2: N/A

**********

4. Have the authors made all data underlying the findings in their manuscript fully available?

The PLOS Data policy

Reviewer #1: No

Reviewer #2: Yes

**********

5. Is the manuscript presented in an intelligible fashion and written in standard English?

Reviewer #1: Yes

Reviewer #2: Yes

**********

Reviewer #1: The topic is interesting.

Thank you for the clarifications provided.

A few comments regarding the answers provided and to improve the quality of your work:

1. I was unable to access the supplementary table S1 (line 146) (which is part of the additional information to be provided in the appendix).

2. Lines 3-4: We sought to explore participant perceptions of a patient-centered model aimed at enhancing care engagement through ‘dynamic choice’ treatment services for mobile individuals.

What distinction do you make between "Participant perspectives" (used in the title of your research) and "Participant perceptions" (used here in line 3)?

I believe the suggestion I made to you addresses this point and emphasizes the importance of maintaining the parallel structure and the appropriate word choice. I note a fundamental difference between perceptions and perspectives, unless I am mistaken.

3. Lines 104-105: I think It is necessary and preferable to provide details within this manuscript, rather than referencing another article, to ensure precision and clarity. This would be the ideal time to fully explain the type of your study and its specific characteristics.

Best regards, and may you find the observations helpful.

Reviewer #2: Dear Author ,

Thank you for this very important study of great importance and relevance ,especially, in sub-saharan Africa.

You have responded to my concerns appropriately .

Simple observation and comment about N and n as in the manuscript attached .

Review this in the Abstract, tables and wherever applicable in the manuscript.

Best of Luck

**********

what does this mean?). If published, this will include your full peer review and any attached files.

If you choose “no”, your identity will remain anonymous but your review may still be made public.

**Do you want your identity to be public for this peer review?**  For information about this choice, including consent withdrawal, please see our Privacy Policy

Reviewer #1: **Yes:** KETEHOUNDJE Désiré

Reviewer #2: **Yes:** Adeloye Amoo Adeniji (MBBS; MMed; FCFP; FACRRM)

**********

[NOTE: If reviewer comments were submitted as an attachment file, they will be attached to this email and accessible via the submission site. Please log into your account, locate the manuscript record, and check for the action link "View Attachments". If this link does not appear, there are no attachment files.]

To ensure your figures meet our technical requirements, please review our figure guidelines: https://journals.plos.org/plosone/s/figures

You may also use PLOS’s free figure tool, NAAS, to help you prepare publication quality figures: https://journals.plos.org/plosone/s/figures#loc-tools-for-figure-preparation.

NAAS will assess whether your figures meet our technical requirements by comparing each figure against our figure specifications.

Attachment Submitted filename: Research manuscript.pdf

# Author comment: Author response to Decision Letter 2

**DOI:** 10.1371/journal.pone.0356605.r004

22 Jun 2026

Please see the attached file; below are the contents:

**

Reviewer 1 Comments to the Author

**

R1: Thank you for the clarifications provided.

A few comments regarding the answers provided and to improve the quality of your work:

1. I was unable to access the supplementary table S1 (line 146) (which is part of the additional information to be provided in the appendix).

Authors: Thank you for bringing this to the editor’s attention. I am on the other side of the platform’s interface, and thus am uncertain how to help.

2. Lines 3-4: We sought to explore participant perceptions of a patient-centered model aimed at enhancing care engagement through ‘dynamic choice’ treatment services for mobile individuals.

What distinction do you make between "Participant perspectives" (used in the title of your research) and "Participant perceptions" (used here in line 3)?

I believe the suggestion I made to you addresses this point and emphasizes the importance of maintaining the parallel structure and the appropriate word choice. I note a fundamental difference between perceptions and perspectives, unless I am mistaken.

Authors: Thank you for noting this. Because we are not presenting a phenomenological account of participants’ experiences, we have use the two terms synonymously, focusing on the overlap in semantic range shared by the two terms.

3. Lines 104-105: I think It is necessary and preferable to provide details within this manuscript, rather than referencing another article, to ensure precision and clarity. This would be the ideal time to fully explain the type of your study and its specific characteristics.

Authors: Thank you for noting this. In the revised manuscript we submitted, we did elaborate on those components of the main study essential to understanding the qualitative component, as follows: “Briefly, its seven components included provision of a “travel pack” containing a travel checklist (with mobility coordinator phone number) to remind participants of what to pack when travelling, discreet ART packing options, and an emergency supply of 14 extra ART pills; a mobility coordinator who would ask participants about their travel plans at each visit and coordinate care with them accordingly; “hotline” phone and SMS access to reach the mobility coordinator as needed; extended refills up to 6 months; community-based delivery of medication for persons unable to return to clinic; assistance in navigating out-of-community ART refills; and transfers of care to out-of-community HIV clinics (when out-migrating without a plan to return) (8). Viral load counselling for participants was also a core component of the SEARCH-SAPPHIRE patient-centred model (22, 35). Control arm participants received usual care and follow-up delivered by the Ministries of Health in the respective countries.”

Additionally, in the methods section we earlier indicated what sort of trial the main study was: “the SEARCH Collaboration designed a differentiated, patient-centred clinical care model intervention to address the needs of mobile participants with HIV.”

R1: Best regards, and may you find the observations helpful.

Authors: Thank you for your close read of the manuscript. Your observations have overall improved the paper.

**

Reviewer 2 Comments to the Author

**

R2: Thank you for this very important study of great importance and relevance , especially, in sub-saharan Africa. You have responded to my concerns appropriately .

Simple observation and comment about N and n as in the manuscript attached .

Review this in the Abstract, tables and wherever applicable in the manuscript.

Authors: Thank you for this note. Because this is a qualitative paper using a subset of the main trial’s number of participants (where quantitative differentiation between N and n is vital for clariy), we have chosen to use N for each separate sample of the qualitative study. We justify this because the two samples in the qualitative study are differentiated by those who received the study components (or control care) and those who delivered it. We feel that using N for both would imply that the providers also enrolled in the main trial as participants. We have therefore kept majuscule N for the purposes of this paper.

**

Authors: Again, we appreciate the helpful comments provided by the reviewers and editor.

Attachment Submitted filename: DCT Main Qual Paper 2nd Response to Reviewers 2026-06-16.docx

# Peer review: Decision Letter 2

**DOI:** 10.1371/journal.pone.0356605.r005

**Authors:** Richard Makurumidze

6 Aug 2026

Participant perspectives on a person-centred care model for mobile individuals in HIV care from the SEARCH Dynamic Choice Treatment trial in rural Kenya and Uganda

PONE-D-25-64298R2

Dear Dr. Johnson-Peretz,

We’re pleased to inform you that your manuscript has been judged scientifically suitable for publication and will be formally accepted for publication once it meets all outstanding technical requirements.

Within one week, you’ll receive an e-mail detailing the required amendments. When these have been addressed, you’ll receive a formal acceptance letter and your manuscript will be scheduled for publication.

An invoice will be generated when your article is formally accepted. Please note, if your institution has a publishing partnership with PLOS and your article meets the relevant criteria, all or part of your publication costs will be covered. Please make sure your user information is up-to-date by logging into Editorial Manager at Editorial Manager® and clicking the ‘Update My Information' link at the top of the page. For questions related to billing, please contact billing support.

If your institution or institutions have a press office, please notify them about your upcoming paper to help maximize its impact. If they’ll be preparing press materials, please inform our press team as soon as possible -- no later than 48 hours after receiving the formal acceptance. Your manuscript will remain under strict press embargo until 2 pm Eastern Time on the date of publication. For more information, please contact onepress@plos.org.

Kind regards,

Richard Makurumidze

Academic Editor

PLOS One

Additional Editor Comments (optional):

Reviewers' comments:

Reviewer's Responses to Questions

**Comments to the Author**

Reviewer #1: All comments have been addressed

Reviewer #2: All comments have been addressed

**********

2. Is the manuscript technically sound, and do the data support the conclusions?

Reviewer #1: Yes

Reviewer #2: Yes

**********

3. Has the statistical analysis been performed appropriately and rigorously?

Reviewer #1: Yes

Reviewer #2: N/A

**********

4. Have the authors made all data underlying the findings in their manuscript fully available?

The PLOS Data policy

Reviewer #1: Yes

Reviewer #2: Yes

**********

5. Is the manuscript presented in an intelligible fashion and written in standard English?

Reviewer #1: Yes

Reviewer #2: Yes

**********

Reviewer #1: The answers given are adequate. I have no further observations.

Reviewer #2: Thank you for the round of review.

I think my major concerns had been satisfactorily addressed.

The issue of N and n also depend's on publishing Journal's requirement and i hope the Editor will use his discretion in this regard.

Good luck

**********

what does this mean?). If published, this will include your full peer review and any attached files.

If you choose “no”, your identity will remain anonymous but your review may still be made public.

**Do you want your identity to be public for this peer review?**  For information about this choice, including consent withdrawal, please see our Privacy Policy

Reviewer #1: **Yes:** KETEHOUNDJE Désiré

Reviewer #2: **Yes:** Adeloye Amoo Adeniji (MBBS;MMed; FCFP; FACRRM)

**********

# Editor report: Acceptance letter

**DOI:** 10.1371/journal.pone.0356605.r006

**Authors:** Richard Makurumidze

PONE-D-25-64298R2

PLOS One

Dear Dr. Johnson-Peretz,

I'm pleased to inform you that your manuscript has been deemed suitable for publication in PLOS One. Congratulations! Your manuscript is now being handed over to our production team.

At this stage, our production department will prepare your paper for publication. This includes ensuring the following:

* All references, tables, and figures are properly cited

* All relevant supporting information is included in the manuscript submission,

* There are no issues that prevent the paper from being properly typeset

You will receive further instructions from the production team, including instructions on how to review your proof when it is ready. Please keep in mind that we are working through a large volume of accepted articles, so please give us a few days to review your paper and let you know the next and final steps.

Lastly, if your institution or institutions have a press office, please let them know about your upcoming paper now to help maximize its impact. If they'll be preparing press materials, please inform our press team within the next 48 hours. Your manuscript will remain under strict press embargo until 2 pm Eastern Time on the date of publication. For more information, please contact onepress@plos.org.

You will receive an invoice from PLOS for your publication fee after your manuscript has reached the completed accept phase. If you receive an email requesting payment before acceptance or for any other service, this may be a phishing scheme. Learn how to identify phishing emails and protect your accounts at https://explore.plos.org/phishing.

If we can help with anything else, please email us at customercare@plos.org.

Thank you for submitting your work to PLOS One and supporting open access.

Kind regards,

PLOS One Editorial Office Staff

on behalf of

Dr. Richard Makurumidze

Academic Editor

PLOS One
